# Establishment of a sensitive TaqMan‐based real‐time PCR assay for porcine circovirus type 3 and its application in retrospective quarantine of imported boars to China

**DOI:** 10.1002/vms3.141

**Published:** 2019-01-14

**Authors:** Chunyan Feng, CaiXia Wang, Yongning Zhang, Fangyuan Du, Zhou Zhang, Fuchuan Xiao, Jianchang Wang, Xiangmei Lin, Shaoqiang Wu

**Affiliations:** ^1^ Institute of Animal Quarantine Chinese Academy of Inspection and Quarantine Beijing China; ^2^ College of Veterinary Medicine Nanjing Agricultural University Nanjing China; ^3^ Center of Inspection and Quarantine Hebei Entry‐Exit Inspection and Quarantine Bureau Shijiazhuang Hebei China

**Keywords:** capsid gene, Porcine circovirus type 3, replicase gene, retrospective quarantine, TaqMan real‐time PCR assay

## Abstract

Porcine circovirus type 3 (PCV3) is a novel pathogen first identified in the United States in 2016. As there is a high possibility that no clinical signs of infection are observed in the host, an accurate and sensitive method is needed for quarantine on numerous live pigs especially for international pig trade. In this study, a TaqMan‐based real‐time PCR assay specifically for PCV3 was established without cross‐reactions with other non‐targeted pig viruses. The sensitivity of the current approach is about 1.5 × 10^1 ^copies *μ*L^−1^ plasmid DNA while the sensitivity of the conventional PCR is about 1.5 × 10^2^ copies *μ*L^−1^ plasmid DNA. Further, this assay was applied in the retrospective quarantine on serum samples of 601 commercial live boars imported to China from the United States, France and the United Kingdom from 2011 to 2017. The results revealed that PCV3 could be detected positive in the commercial boars imported from the United States and the above‐mentioned western European countries and phylogenetic study also revealed that viral isolates were grouped with some isolates from Korea and the United States. Our study suggested that PCV3 may be prevalent globally since 2011.

## Introduction

Porcine circovirus type 3 (PCV3) is a type of non‐enveloped and single‐stranded circular DNA virus with 2000 nucleotides (nt) and it was associated with porcine dermatitis and nephropathy syndrome (PDNS), reproductive failure cardiac and multi‐systemic inflammation and respiratory disease complex (Palinski *et al*. 2016). Three open reading frames (ORFs) were identified and named as capsid (CAP) gene, replicase (REP) gene and ORF3 gene (unknown function). Together with Porcine circovirus type 1 (PCV1) and Porcine circovirus type 2 (PCV2), PCV3 belongs to the genus circoviruses in the family of circoviridae.

PCV3 was identified in the United States in 2016 as a pathogen agent associated with pig diseases. Then, the PCV3 infections in the pigs were reported in China, Korean, Poland and Italy (Ku *et al*. 2017; Shen *et al*. 2017; Kwon *et al*. 2017; Stadejek *et al*. 2017; Faccini *et al*. 2017). Studies also showed that PCV3 infected pigs may show no clinical signs (Zheng *et al*. 2017). Some reports showed that there is a chance of co‐infection of PCV3 and PCV2 (Wang *et al*. 2017).

In China, swine industry plays a significant role in national economics. From time to time, China has to improve the breeding of pigs by importing live boars from the United States and European countries. To date, very few data were available on the virus global transmission or on its origin. In this study, a sensitive TaqMan real‐time PCR method was established. This method was applied in retrospective quarantine of the PCV3 DNA in the serum samples collected from 601 boars imported to China from the United States, France and the United Kingdom between 2011 and 2017. Our study would offer a new clue to global spreading of PCV3.

## Materials and methods

For real‐time PCR method development, the whole genome of PCV3 was synthesized artificially by Takara (Takara, China) based on the reference sequences available from GenBank (No. KT869077). Then, the genome of PCV3 was cloned into pMD‐19T Vector followed by the process of amplification, purification and quantification using a ND‐2000c spectrophotometer (NanoDrop, USA). The copy numbers were calculated using the formula in Ref. Yun *et al*. (2006). Considering that field samples would contain the inhibitors that are likely to be present in the biological samples, we performed DNA extraction on 500 *μ*L of negative serum using DNA extraction Kit. The product finally dissolved in 200 *μ*L of H_2_O as dilution buffer. Tenfold dilutions of the PCV3‐pMD‐19T, ranging from 10^8^ to 10^0^ copies *μ*L^−1^ were prepared in the dilution buffer. Aliquots of each dilution were stored at −70°C until use.

The aimed REP fragment amplified by this TaqMan PCR assay was blasted first. The primers and probes for PCV3 TaqMan PCR assays were then designed, using the known sequences of PCV3 strains from GenBank. The conventional PCR primers used in this study were designed in the same way based on capsid gene(Table 1). PCV2, Pseudorabies virus (PRV) and Porcine parvovirus (PPV) were used as control to verify the specificity of the primers and probe of the TaqMan PCR assay. The DNA samples of these viruses were offered by Beijing DaBeiNong Technology Group Co., Ltd.

**Table 1:** Nucleotide sequences of primers and probe used in the TaqMan real‐time PCR assay and in conventional PCR in this study

Name	Sequence 5′– 3′	Nucleotideposition	Purpose
Probe	FAM‐ AGTAATGTTGTACCGGAGGAGTGG‐BHQ1	960–983	TaqMan real time PCR
Forward primer	TGTGGCTAAGGTATTATATATTAC	935–958	
Reverse primer	TCTCCATGTCTTTCTTTACC	1064–1083	
PCV3U1	ACTTGTAACGAATCCAAACTTC	1347–1368	Conventional PCR
PCV3L1	TGGCACGCCAACCACTTCATTA	1782–1803	

Real‐time PCR assay was performed with iQ5 instrument (Bio‐Rad, Hercules, USA). TaKaRa Premix Ex Taq (Probe qPCR) of 2× concentration (Cat. No. RR390A) was used during the test and it contains TaKaRa Ex Taq HS,dNTP Mixture,Mg^2+^ and Tli RNaseH. We used TaqMan Probe in the current standardized protocol. The oligo sequence 5’‐FAM‐AGTAATGTTGTACCGGAGGAGTGG‐BHQ1‐3’ has been labelled with reporter FAM (Carboxfluorescein) at 5’ end and quencher BHQ1 at 3’ end. This probe was used at a final concentration of 0.25 *μ*mol L^−1^. The primers for qPCR as mentioned in Table 1 were used at a final concentration of 0.75 *μ*mol L^−1^. The DNA extracted from serum samples was diluted fivefold and used as a template and the samples’ amplification curves were obtained. Each reaction was performed in triplicates with negative control (water as template) and positive control (plasmids template). The cycling conditions are pre‐incubation at 95°C for 30 s, initial denaturation at 95°C for 5 s and annealing at 57°C for 30 s. To prevent cross contamination, <30 samples were detected using this assay in one performance. For further confirmation, the real‐time PCR products were visualized on a 1.5% (w/v) agarose gel.

To analyse the sensitivity of the assay, 10‐fold serial dilutions of plasmid DNA starting with a concentration of 1.5 × 10^8^ copies *μ*L^−1^ were prepared. They were used as standards for obtaining a standard curve. The threshold cycle (*C*_t_) and the PCR efficiency of the amplification were calculated by LinRegPCR program (version11.0, downloaded from http://LinRegPCR.HFRC.nl). Conventional PCR was also performed to compare with the TaqMan PCR.

To determine the reproducibility of the real‐time PCR, 10^2^–10^6^ copies *μ*L^−1^ of plasmid DNA were tested in three different times and in triplicate to determine the coefficient of variation (CV). The intra‐ and inter‐assay CVs for *C*_t_ values were calculated.

Serum samples collected from 2011 to 2017 were provided by Hebei, Shanghai and Hunan Entry‐Exit Inspection and Quarantine Bureau of China, from commercially imported live boars from the United States, the United Kingdom and France. Based on different time, totally six batches of samples were quarantined in this study (Table 2). All the commercial boars were recorded in healthy state throughout the selection process of the international trade. Blood samples were collected according to standard operating procedures: approximately 3 or 5 days after the commercial boars landed and arrived at the isolation premise. The sera were then separated and divided for further detection and the redundant sera were stored at −70°C.

**Table 2:** Detection results of PCV3 in the serum samples of boars imported from France, the United Kingdom and the United states between 2011 and 2017

Year	Country	Sample numbers	Positive ratio (%)	Positive numbers
2011	UK	26	7.69	2
2011	France	89	7.87	7
2011	United states	37	10.8	4
2012	France	50	4.00	2
2014	UK	100	6.00	6
2017	United states	299	11.04	33

Total DNA was extracted from 100 *μ*l serum using Wizard ^®^Genomic DNA Purification Kit, (Promega, USA) and then dissolved in 40 *μ*L ddH_2_O. To prevent the cross contaminations, the process was done by groups and each group consists of less than 30 samples.

Further, the TaqMan Real‐Time PCR assay was applied to the retrospection of the blood serum of commercially imported live boars during the years 2011–2017 mentioned above. Totally 601 samples were detected by the assay. Samples with *C*_t_ values ≤40 and with typical amplification curves were considered positive (Staggs *et al*. 2013; Tatti *et al*. 2011; Lakshmi *et al*. 2018). The study also includes verification of the assay results: the sample dilutions that marked amplification and part of no amplification were detected by droplet digital PCR (ddPCR).

To amplify the partial capsid gene from PCV3 positive samples, two rounds of conventional PCR were performed using the high fidelity Pfu polymerase. Briefly, after the first round PCR, product was separated by 1.5% agarose gel and the segment around 457 bp was cut for further purification and enrichment although there was no visualization of the specific band. To prevent the cross contaminations, the electrophoresis chamber was washed carefully and the running buffer was changed for every performance. The purified and enriched DNA samples were used as templates for the second round PCR. After that, the PCR product was separated on a 1.5% agarose gel, then purified, cloned to pGEM‐T vector (Promege, USA) and sequenced.

Totally 13 partial capsid genes of PCV3 were sequenced (two samples from each batch in addition to three samples from the 2017 US batch) and compared with 29 selected capsid gene sequences downloaded from NCBI database. Multiple sequence alignments and phylogenetic analysis were performed using MEGA 7. The evolutionary history was inferred by using the Maximum Likelihood method based on the Tamura‐Nei model (Tamura & Nei 1993). Evolutionary analyses were conducted by MEGA7 (Kumar *et al*. 2016).

## Results

**Figure 1:**
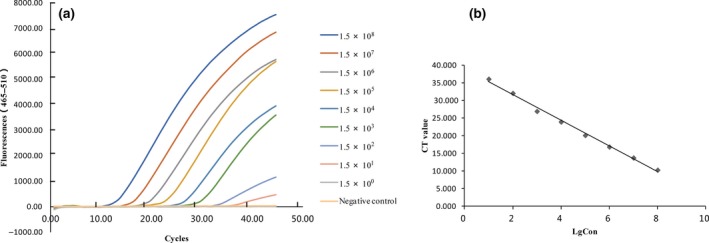
The sensitivity detection and Standard curves of the Taqman PCR assay. (a) Representative amplification chart of sensitivity of the Taqman PCR assay. The sensitivity of the TaqMan real‐time PCR assay was 1.5 × 10^1^ copies *μ*L^−1^ of plasmid DNA. (b) Standard curve of the real‐time PCR based on serial dilutions of the plasmid DNA. Mean threshold cycle *C*_t_ values from three replicates (*y*‐axis) versus logarithmic concentrations of plasmid copies (*x*‐axis) are plotted.

Primers and probe sequences, length of PCR products and their positions are shown in Table 1. The standard curve was generated with a range of 10^8^–10^0^ plasmid copies *μ*L^−1^ (Fig. 1a). Using LinRegPCR method, the *C*_t_ value was estimated and the reaction efficiency of this assay was 98.5%. The linear relationship between Log concentration of plasmid copies *μ*L^−1^ and *C*_t_ value is *y* = −3.6481x + 38.933 (Fig. 1b). The assay was linear over a 10 dilution range of template DNA with *R* ^2^ values (square of the correlation coefficient) of 0.995. In this study, 1.5 × 10^1 ^copies *μ*L^−1^ of plasmid DNA could be detected by the TaqMan real‐time PCR assay (Fig. 1). By comparison, 1.5 × 10^2^ copies *μ*L^−1^ of plasmid DNA instead of 1.5 × 10^1 ^copies *μ*L^−1^ could be detected by conventional PCR (Appendix S1).

For specificity study, the blast analysis of the aimed replicase gene showed that except for the PCV3 strains, no homologue sequences of other virus were detected. In the specificity experiment, it is shown that PRV, PPV, PCV2 and tissue sample did not yield any positive result (Appendix S2), corresponding to bioinformatics analysis results mentioned above, demonstrating the high specificity of the assay.

For the reproducibility of the real‐time PCR, 10^2^–10^6^ copies *μ*L^−1^ of PCV3‐ pMD‐19T were tested in the real‐time PCR, the intra‐assay CVs were 5.01, 1.91, 3.21, 3.49 and 1.29%, respectively. The inter‐assay CVs were 1.13, 2.82, 1.84, 2.20 and 4.03%, respectively.

For retrospection of the PCV3 virus in commercially imported live boars from the United States, the United Kingdom and France between 2011 and 2017, six batches of the total 601 samples were quarantined. The samples were considered positive when the *C*_t_ values were ≤ 40 and they showed typical amplification curves and the TaqMan PCR products of them showed specific band around 149 bp (Appendix S3). As shown by the results, positive PCV3 samples were detected in all the six batches and there are totally 53 positive samples (Table 2). For the samples from the United States, 4 out of 37 were detected positive (samples of 2011) and 33 out of 299 were showed positive (samples of 2017). For the samples from the United Kingdom, 2 out of 26 were detected positive (samples of 2011) and 6 out of 100 were detected positive (samples of 2014). For the samples from France, 7 out of 89 were detected positive (samples of 2011) and 2 out of 50 were detected positive (samples of 2012).

The minimum positive rate of the six batches was 4.00%, corresponding to the samples from France in 2012. The highest positive rate was 11.04%, corresponding to the samples from the United States in 2017. The highest copies of PCV3 detected in the blood sample were the 2017 samples from the United States and the value was as high as 656 copies *μ*L^−1^ of viral DNA.

**Figure 2:**
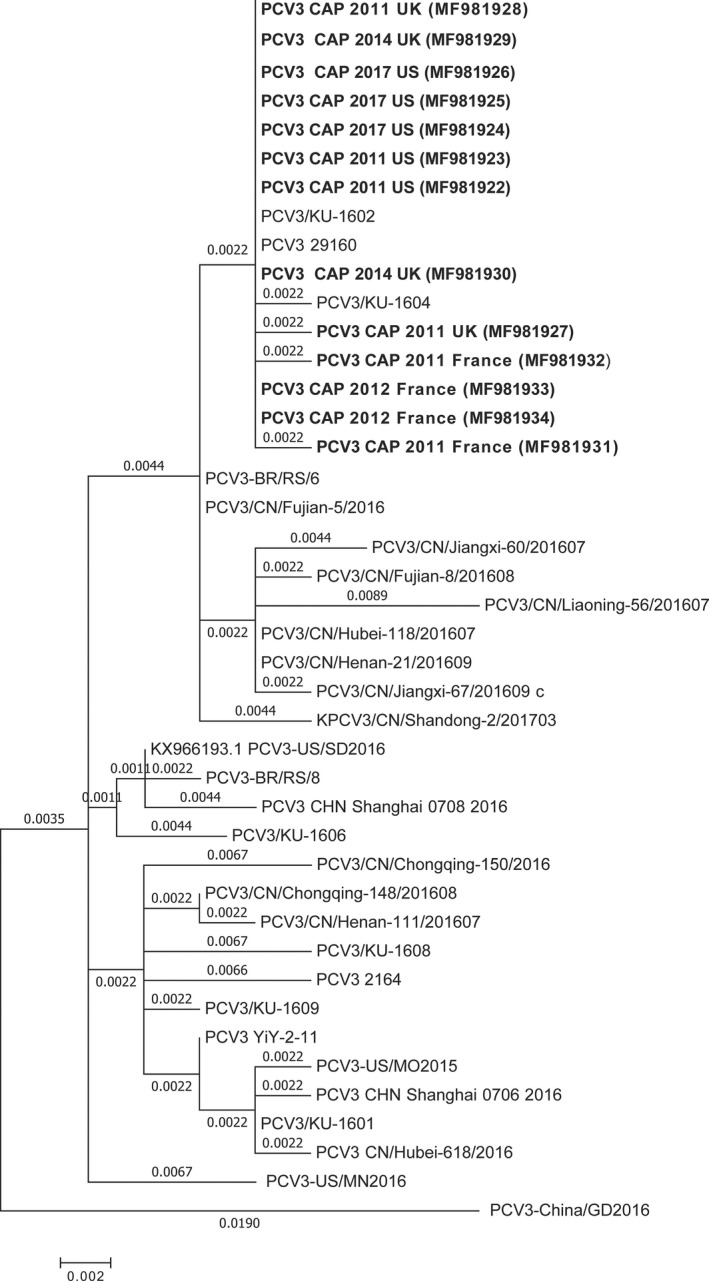
The phylogenetic analysis of partial cap gene of PCV3 sequenced in this study. The isolates with black bold indicated the partial cap genes sequenced in this study. Scale bars indicated nucleotide substitutions per site.

Sequencing of 13 PCR products (457 bp segment from capsid gene) of samples from six batches showed that all of them were partial capsid genes of PCV3 (Genbank No. MF981922 – No. MF981834, Appendix S4). The phylogenetic analysis results exhibit obvious divergence of partial capsid gene of PCV3 (Fig. 2). Interestingly, all sequence of partial capsid gene identified in this study were classified into the same clade with PCV3/KU‐1602 (KY996338, isolate in Korea), PCV3 29160 (KT869077, isolate in United States) and PCV3/KU‐1604 (KY996340, isolate in Korea). Of note, three sequences of capsid gene from the United Kingdom and five sequences from the United States detected by this study showed 100% identity to PCV3/KU‐1602 from Korea (Kwon *et al*. 2017) and PCV3 29160 from the United States (Palinski *et al*., 2016).

## Discussion

In this study, a real‐time PCR assay for sensitive detection of PCV3 was established. Plasmid DNA was used to construct the standard curve. It should be noticed that the field blood samples, which are likely to contain the inhibitors are more complex than plasmid DNA and are harder to detect. When constructing the standard curve, we prepared the dilution buffer by treating negative serum with DNA extraction kit and we used such dilution buffer to dilute the plasmid DNA. For the same reason, the DNA copies of the field samples are supposed to be higher than the plasmid samples, given that they have the same *C*_t_ value. So, *C*_t_ value ≤ 40 was considered to be positive for field samples. This criterion for *C*_t_ value was also adopted by other groups (Staggs *et al*. 2013; Tatti *et al*. 2011; Lakshmi *et al*. 2018).

An improper baseline setting may severely affect the estimated PCR efficiencies and will thus increase the variability as well as the bias in the detected copy number of aimed DNA (Ruijter *et al*. 2009). To solve this problem, LinregPCR method was employed to obtain the threshold cycle (*C*_t_) and to calculate the efficiency of this assay. After the reaction‐system refinement i.e., optimizing the ratio of primer and probe, selecting a proper buffer and changing quencher from TAMARA to BHQ1, the efficiency of the standard curve had been improved to 98.5% in this study.

Before this study, several PCR assays had been developed for the detection of PCV3, including one conventional PCR and two real‐time PCR. Compared to the other PCR assays, our assay shows advantages. As low as 1.5 × 10^1^ copies *μ*L^−1^ concentrations of plasmid DNA and five copies of viral DNA from serum sample were able to be detected by this assay (Fig. 1 and Table 2). We carried out two rounds of conventional PCR on the same samples and the following sequence of the PCR products confirmed the positivity of the results. ddPCR was also performed and confirmed the positive samples. Thus, our method is able to detect this virus with low copies, which has no clinical symptoms and is hard to be detected with one round conventional PCR method. In comparison, the real‐time PCR assay established by Wang *et al*. (2017) was could detect 10^2 ^copies *μ*L^−1^ instead of 10^1^copies *μ*L^−1^ plasmids DNA. We did not compare our method with another important real‐time PCR established by Palinski *et al*. (2016). due to technical difficulties in synthesis of their designed probe. Based on the comparisons mentioned above, the assay was suitable for quarantine of numerous serum samples from live pigs in healthy state, especially for port‐related implementation of serum quarantine.

Since the first reported PCV3 virus in the United States, most following reports were focusing on prevalence and the genomic characteristic of the virus. This virus was subsequently reported in Asian countries like China and Korea. In China, this virus was reported in more than six provinces, including Hebei, Shandong, Liaoning, Fujian, Hubei, etc. The earliest case could be retrospective to the clinical sample collected in 2014 (Ku *et al*. 2017; Fan *et al*. 2017; Zheng *et al*. 2017). Some isolates from China shared high identity with PCV3/29160 and PCV3/2164 from USA (Shen *et al*. 2017). The CAP protein of Korean strains shared over 98% identities to that of the US strains (Kwon *et al*. 2017). No PCV3 was reported in European countries except Poland and Italy recently (Stadejek *et al*. 2017; Faccini *et al*. 2017). The first case of infection found in Poland was in 2015 and the genetic sequence analysis of partial ORF2 showed very high identity to PCV3‐US/SD2016 (Stadejek *et al*. 2017). In our study, we found that all batches of live pigs imported to China for breeding were carrying PCV3 virus. Of note, our research first showed that PCV3 existed in European countries like France and the United Kingdom and the time can be traced back to the year 2011. During our study, the virus was detected positive again in the live boars imported from France in the year 2012 and in the live boars imported from the United Kingdom in the year 2014. The partial capsid genes amplified by our group showed that they possess high identity to PCV3/29160 (Fig. 2).

We also noticed that the number of PCV3 DNA copies of the boars was low in this study. The overall number of DNA copies of PCV3 in boars that from France is 5 –95 copies *μ*L^−1^, which is lower than the Unite states (5–656 copies *μ*L^−1^) and the United Kingdom (6–111 copies *μ*L^−1^). The overall number of DNA copies of PCV3 in boars from the United States is relatively higher than the other two countries and the maximum number is 656 copies *μ*L^−1^. Low copies of virus may be the reason why the commercial boars examined in this study were in healthy state without any clinic symptoms. This is consistent with the report that PCV3 infected host may show no clinical signs of infection (Zheng *et al*. 2017).

It was reported that PCV3 was detected in fetuses and it indicated that PCV3 could spread by vertical transmission (Ku *et al*. 2017). In this study, sequencing of the partials capsid gene revealed a high homology among the investigated strains and they also showed high homology with the fragment of CAP PCV3/29160. For these commercial boars were imported to China for breeding pigs, it was not hard to infer that some prevalence strains of PCV3 in China maybe originate from USA or European countries. For the limited knowledge of the final location of these imported commercial boars, the transmission and its spreading in China still need further investigation. Probably due to the low copies of the viral DNA in the serum in this study, it was very hard to amplify the full‐length genome and more effort is needed on this task.

In conclusion, a TaqMan real‐time PCR assay has been developed based on replicase gene of PCV3. Based on this method, our study is the first to report PCV3 found in samples from the United Kingdom and France. Moreover, we found that one origin of the prevalent strain PCV3 in China was possibly from the commercial boars imported from the United States or the European countries, such as the United Kingdom and France.

## Source of funding

This work was supported by the National Key R&D Program of China (2016YFD0501105 and 2016YFC1201603‐X) from the Ministry of Science and Technology of China.

## Conflicts of interest

The authors declare that they have no competing interests.

## Ethical statement

The authors confirm that the ethical policies of the journal, as noted on the journal's author guidelines page have been adhered to and the appropriate ethics review committee approval has been received. The US National Research Council's guidelines for the Care and Use of Laboratory Animals were followed.

## Contribution

CYF, CXW and SQW designed the study. CYF, CXW, FCX, YNZ, FYD, and ZZ performed the laboratory work. CYF, CXW, SQW and XML analyzed the results and prepared the draft manuscript. JCW participated in sample collection. All authors collaborated in the revision and approval of final paper.

## Supporting information


**Appendix S1.** The sensitivity Compare of the TaqMan real time PCR and conventional PCR.
**Appendix S2.** Determination of the specificity of the TaqMan real‐time PCR assay.
**Appendix S3.** The representative graphic of one performance of quarantine on 26 serum samples of live boars imported from the United Kingdom in 2011 to China.
**Appendix S4.** Basic information of Sequenced positive samples.
