# Supplementary material for: Establishment of a sensitive TaqMan‐based real‐time PCR assay for porcine circovirus type 3 and its application in retrospective quarantine of imported boars to China
Source: Vet Med Sci. 2019 Jan 14;5(2):168–75. doi: 10.1002/vms3.141 (PMC6498530; doi:10.1002/vms3.141)
Supplement: Supplementary file 1 — Appendix S1. The sensitivity Compare of the TaqMan real time PCR and conventional PCR. Appendix S2. Determination of the specificity of the TaqMan real‐time PCR assay. Appendix S3. The representative graphic of one performance of quarantine on 26 serum samples of live boars imported from the United Kingdom in 2011 to China. Appendix S4. Basic information of Sequenced positive samples. [file VMS3-5-168-s001.docx]

**Appendix documents**

Appendix 1 The sensitivity Compare of the TaqMan real time PCR and conventional PCR.


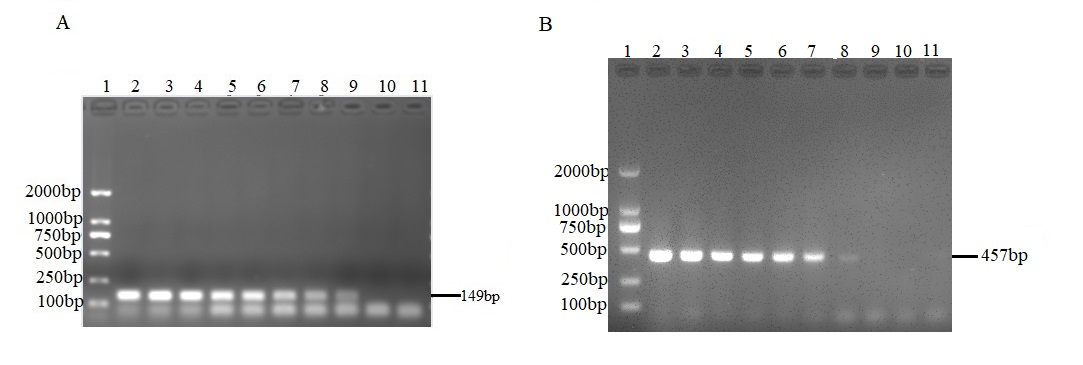


A: The sensitivity of the TaqMan real time PCR assay was 1.5 × 10^1^ copies/μl plasmid DNA. line 1: Marker; line 2-line 10: TaqMan PCR products of 10^8^ copies/μl – 10^0^ copies/μl plasmid DNA as template; line 11: negative control.

B: The sensitivity of conventional PCR assay was 1.5 × 10^2^ copies/μl plasmid DNA. Line 1：Marker；line 2- line 10：PCR products of 10^8^ copies/μl – 10^0^ copies/μl plasmid DNA as template; Line 11: negative control.

Appendix 2 Determination of the specificity of the TaqMan real-time PCR assay.


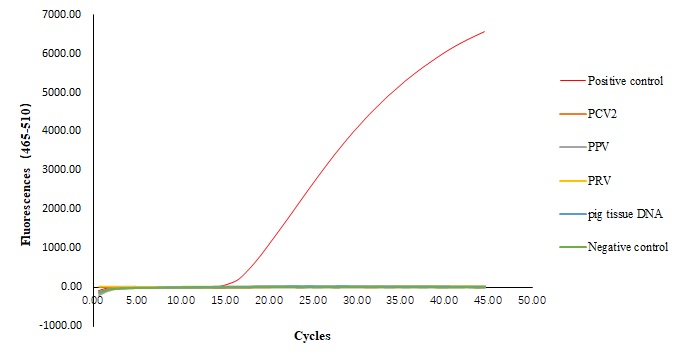


Appendix 3 The representative graphic of one performance of quarantine on 26 serum samples of live boars imported from the United Kingdom in 2011 to China.


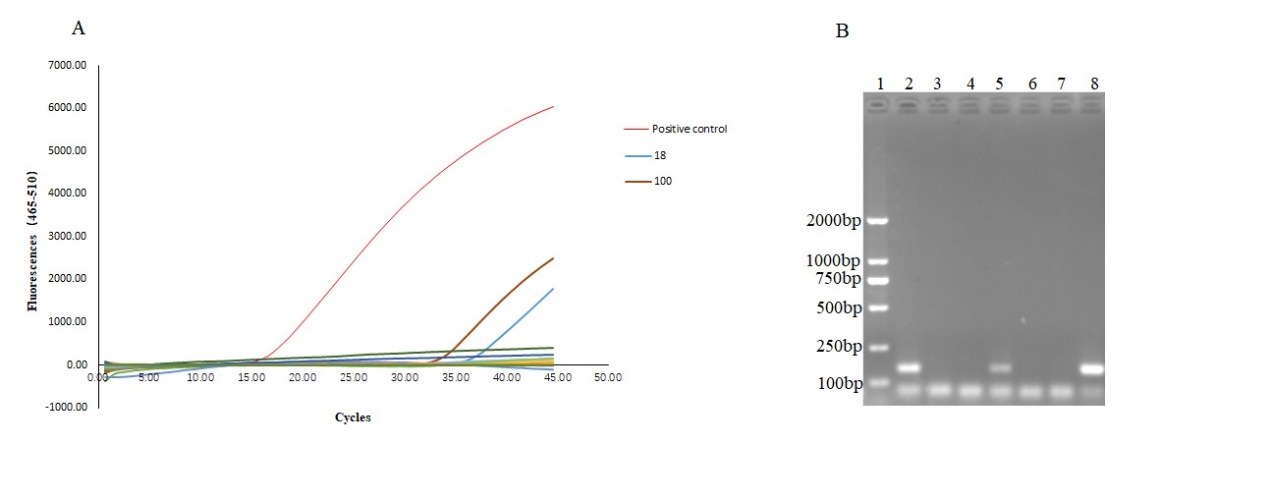


A: The representative amplification chart of the TaqMan real-time PCR. Sample No. 100 and Sample No.18 were showed to have typical amplification curves. B: The electrophoresis results of positive products of the TaqMan real-time PCR. Line 1: maker; line 2: sample No. 100; line 3: negative sample; line 4: negative sample; line 5: sample No.18; line 6: negative sample; line7: negative control; line 8: positive control.

Appendix 4 Basic information of Sequenced positive samples.

| Sample  Name | country | Year | Ct value | Viral DNA copies/μl | Genbank No. |
| --- | --- | --- | --- | --- | --- |
| 100 | UK | 2011 | 32.22 | 104 | MF981928 |
| 18 | UK | 2011 | 36.56 | 7 | MF981927 |
| 46 | France | 2011 | 33.25 | 54 | MF981932 |
| 222 | France | 2011 | 32.36 | 95 | MF981931 |
| 37 | United states | 2011 | 30.21 | 369 | MF981922 |
| 275 | United states | 2011 | 32.23 | 103 | MF981923 |
| 1-043 | France | 2012 | 35.22 | 16 | MF981933 |
| 1-063 | Fance | 2012 | 35.55 | 13 | MF981934 |
| A-38 | UK | 2014 | 32.11 | 111 | MF981929 |
| B 039 | UK | 2014 | 34.55 | 24 | MF981930 |
| 214 | United states | 2017 | 29.3 | 656 | MF981924 |
| 716 | United states | 2017 | 30.56 | 296 | MF981925 |
| 114 | United states | 2017 | 29.88 | 455 | MF981926 |
